# Increased aquaporin 1 water channel expression inhuman brain tumours

**DOI:** 10.1038/sj.bjc.6600512

**Published:** 2002-09-09

**Authors:** S Saadoun, M C Papadopoulos, D C Davies, B A Bell, S Krishna

**Affiliations:** Department of Infectious Diseases, St. George's Hospital Medical School, Cranmer Terrace, Tooting, London SW17 0RE, UK; Department of Neurosurgery, Atkinson Morley's Hospital, Copse Hill, Wimbledon, London SW20 0NE, UK; Department of Anatomy and Developmental Biology, St. George's Hospital Medical School, Cranmer Terrace, Tooting, London SW17 0RE, UK

**Keywords:** adenocarcinoma, brain neoplasms, brain oedema, cytotoxic oedema, glioma, vasogenic oedema

## Abstract

Aquaporin 1 is a water channel protein. There was little aquaporin 1 immunoreactivity in normal brain parenchyma. In astrocytomas, aquaporin 1 was expressed in microvessel endothelia and neoplastic astrocytes. In metastatic carcinomas, aquaporin 1 was present in microvessel endothelia and reactive astrocytes. Aquaporin 1 may participate in the formation of brain tumour oedema.

*British Journal of Cancer* (2002) **21**, 621–623. doi:10.1038/sj.bjc.6600512 www.bjcancer.com

© 2002 Cancer Research UK

## 

Astrocytomas and metastatic carcinomas are the most common brain tumours in adults and are associated with brain oedema, which increases patient morbidity and mortality. The molecular mechanisms responsible for this brain oedema are poorly understood (Papadopoulos *et al*, 2001). A recently discovered family of water channels proteins, called aquaporins (AQPs), may provide a novel molecular explanation for the formation of human brain tumour oedema.

The aquaporins (AQPs) are a family of 10 highly conserved water channel proteins that provide the molecular pathway for water permeability in water-transporting tissues (Verkman and Mitra, 2001). Evidence of human diseases resulting from alterations in AQP gene expression or regulation is limited. Mutations in AQP2 cause nephrogenic diabetes insipidus and AQP0 mutations have been associated with cataract formation (Verkman and Mitra, 2001). However, humans lacking the AQP1 protein appear phenotypically normal (Verkman and Mitra, 2001).

In rodents, only AQP4 and AQP1 are significantly expressed in normal brain. AQP4 is expressed in astrocyte endfeet around microvessels and in the glial limiting membranes (Verkman and Mitra, 2001). AQP1 is expressed in choroid plexus epithelium and may be important in the formation of cerebrospinal fluid (Verkman and Mitra, 2001). In man, there is massive upregulation of AQP4 expression in tumour cells in high-grade astrocytomas and reactive astrocytes around metastatic carcinomas (Saadoun *et al*, 2002). However, little is known about AQP1 expression in normal human brain and human brain tumours.

## MATERIALS AND METHODS

Immunohistochemistry was used to investigate the expression of AQP1 in morphologically normal human brain (*n*=5), low- (*n*=5) and high-grade (*n*=5) astrocytomas and metastatic carcinomas (*n*=5). The study was approved by St. George's Healthcare Ethics Committee. Astrocytomas were classified as low (grades I–II) or high (grades III–IV) grade according to the Daumas-Duport criteria. Non-neoplastic cerebral cortex was obtained from patients who underwent temporal lobectomies for intractable epilepsy (*n*=3) and from the entry sites of ventricular drains (*n*=2). All patients with brain tumours (*n*=15) received dexamethasone, compared with three out of five of those who contributed histologically normal brain tissue. Tissue specimens were fixed in buffered formalin/saline and processed into paraffin wax. Tissue sections (10 μm) were cut and incubated with a polyclonal rabbit anti-AQP1 antibody (AB3065, Chemicon), followed by a goat anti-rabbit biotinylated antibody (Sigma), avidin–biotin–horseradish peroxidase and diaminobenzidine tetrachloride/H_2_O_2_. Sections from each subject were also immunoreacted with a polyclonal rabbit anti-glial fibrillary acidic protein (GFAP) primary antibody (Dako) to aid identification of the cells expressing AQP1. Omitting the primary or secondary antibody abolished staining. Assessment of the sections was performed ‘blind' by two investigators. Kendall's tau-B test was used to quantify the relationship between tied non-parametric qualitative data.

## RESULTS

**Figure 1:**
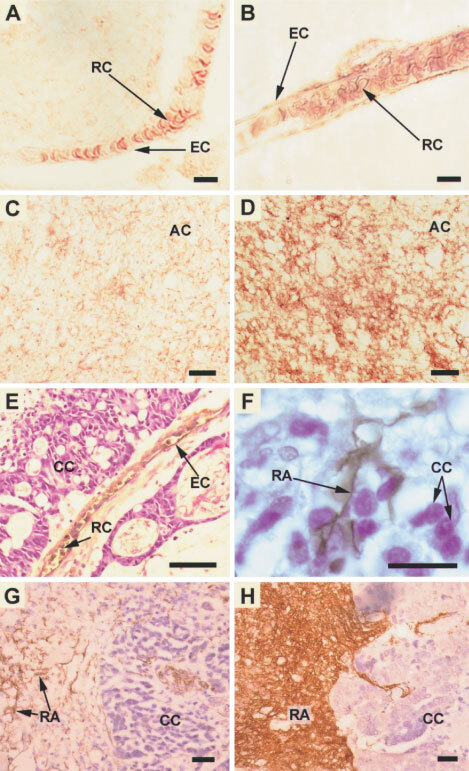
Immunostaining (brown) for (**A**–**G**) AQP1 and (**H**) GFAP with cresyl violet counterstain. (**A**) and (**B**) Microvessels from normal brain tissue. In (**A**), which is typical of most microvessels in normal brain, only the red blood cell membranes stain for AQP1. In a few microvessels in normal brain, such as (**B**), the endothelial cell membranes also stain for AQP1. AQP1 immunoreactivity is upregulated in (**C**) low-grade and (**D**) high-grade astrocytoma. (**E**–**H**) Photomicrographs of carcinoma metastases to brain. In (**E**) the microvessel endothelium and red blood cells immunostain for AQP1. (**F**) Shows an AQP1 immunopositive astrocyte trapped between cancer cells. In (**G**) reactive astrocytes immunostain for AQP1. (**H**) Shows that the cells which express AQP1 also stain for GFAP. Bars=10 μm (**A**,**B**), 90 μm (**C**–**E**), 30 μm (**F**–**H**). AC=astrocytoma cells, CC=carcinoma cells, EC=endothelial cells, RA=reactive astrocytes, RC=red blood cells.

In all specimens AQP1 was detected in erythrocyte membranes. In sections of normal brain, AQP1 immunolabelling appeared as brown deposit over the endothelium of a few (<33%) microvessels (Figure 1B

). There was no immunolabelling of the brain parenchyma (Figure 1A,B). In low-grade astrocytomas, AQP1 immunoreactivity was present in tumour cells, mostly in the region of the cell membrane (Figure 1C). In high-grade astrocytomas, AQP1 immunoreactivity was massively upregulated and distributed throughout the cytoplasm of neoplastic cells (Figure 1D). The amount of AQP1 in astrocytomas strongly correlated with the grade of malignancy (Table 1

**Table 1:**
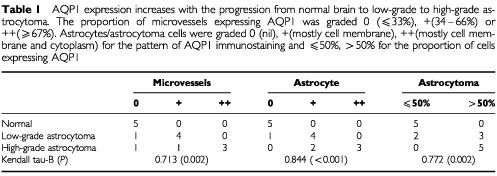
AQP1 expression increases with the progression from normal brain to low-grade to high-grade astrocytoma. The proportion of microvessels expressing AQP1 was graded 0 (⩽33%), +(34–66%) or ++(⩾67%). Astrocytes/astrocytoma cells were graded 0 (nil), +(mostly cell membrane), ++(mostly cell membrane and cytoplasm) for the pattern of AQP1 immunostaining and ⩽50%, >50% for the proportion of cells expressing AQP1

). In all metastatic carcinomas, AQP1 was expressed in microvessel endothelium (Figure 1E) and reactive astrocytes (Figure 1F–H). AQP1 was not detected in cancer cells.

## DISCUSSION

The current results agree with data from a study of AQP1 expression in glioblastoma and breast cancer transplanted into mouse brain (Endo *et al*, 1999). In that study, AQP1 immunoreactivity was found in glioblastoma cells and microvascular endothelial cells, but not normal brain parenchyma or normal microvessel endothelium. AQP1 mRNA levels have recently been shown to be increased in human glioblastoma, compared with normal human brain (Markert *et al*, 2001).

There was little or no AQP1 expression in normal brain microvessel endothelium, which is consistent with its low permeability to many substances. The microvascular endothelium of brain tumours has an impaired blood–brain barrier function (Papadopoulos *et al*, 2001) and the current results suggest that its permeability to water may also be increased. The signals that induce AQP1 expression in the endothelium of brain tumours are unknown, but might include vascular endothelial growth factor, which is produced by tumour cells and is known to increase vascular permeability.

The presence of AQP1 immunoreactivity in both cell membrane and cytoplasm of high-grade astrocytoma cells suggests a high turnover of AQP1 protein. Assuming that AQP1 protein is functional in brain tumours, AQP1 may be contributing to the flow of oedema fluid through the tissue. AQP1 water channel blockers might thus be potent anti-brain tumour oedema agents.

Glucocorticoids are commonly used to reduce brain tumour oedema, but their mechanism of action is unclear. Since all of the tumour patients received dexamethasone, it was not possible to determine the effect of glucocorticoids on AQP1 expression. Interestingly, the AQP1 promoter, sequenced from human erythroleukaemia cells, possesses steroid responsive elements (Moon *et al*, 1997). It is, therefore, possible that the anti-brain tumour oedema action of glucocorticoids is at least partly explained by alterations in AQP1 expression.

Further studies are needed to clarify the mechanisms responsible for the upregulation of AQP1 (present study) and AQP4 (Saadoun *et al*, 2002) expression in brain tumours and the contribution of these water channels to brain tumour oedema. Co-cultures of neurons, astrocytes, endothelial or cancer cells may elucidate the role of cell-cell interactions in the control of water channel expression. Brain tumour models comparing wild-type mice with AQP1 and AQP4 knockout mice may shed light on the contribution of these water channels to brain tumour oedema. High throughput screening of combinatorial libraries and other techniques are underway to identify water channel blockers, which might reduce brain tumour oedema.
